# Systematic Dissection of Coding Exons at Single Nucleotide Resolution Supports an Additional Role in Cell-Specific Transcriptional Regulation

**DOI:** 10.1371/journal.pgen.1004592

**Published:** 2014-10-23

**Authors:** Ramon Y. Birnbaum, Rupali P. Patwardhan, Mee J. Kim, Gregory M. Findlay, Beth Martin, Jingjing Zhao, Robert J. A. Bell, Robin P. Smith, Angel A. Ku, Jay Shendure, Nadav Ahituv

**Affiliations:** 1 Department of Bioengineering and Therapeutic Sciences, University of California San Francisco, San Francisco, California, United States of America; 2 Institute for Human Genetics, University of California San Francisco, San Francisco, California, United States of America; 3 Department of Life Sciences, Ben-Gurion University at the Negev, Beer-Sheva, Israel; 4 Department of Genome Sciences, University of Washington, Seattle, Washington, United States of America; 5 Key Laboratory of Advanced Control and Optimization for Chemical Processes of the Ministry of Education, East China University of Science and Technology, Shanghai, China; 6 Department of Neurological Surgery, University of California San Francisco, San Francisco, California, United States of America; 7 Biomedical Sciences (BMS) Graduate Program, University of California San Francisco, San Francisco, California, United States of America; Stanford University School of Medicine, United States of America

## Abstract

In addition to their protein coding function, exons can also serve as transcriptional enhancers. Mutations in these exonic-enhancers (eExons) could alter both protein function and transcription. However, the functional consequence of eExon mutations is not well known. Here, using massively parallel reporter assays, we dissect the enhancer activity of three liver eExons (*SORL1* exon 17, *TRAF3IP2* exon 2, *PPARG* exon 6) at single nucleotide resolution in the mouse liver. We find that both synonymous and non-synonymous mutations have similar effects on enhancer activity and many of the deleterious mutation clusters overlap known liver-associated transcription factor binding sites. Carrying a similar massively parallel reporter assay in HeLa cells with these three eExons found differences in their mutation profiles compared to the liver, suggesting that enhancers could have distinct operating profiles in different tissues. Our results demonstrate that eExon mutations could lead to multiple phenotypes by disrupting both the protein sequence and enhancer activity and that enhancers can have distinct mutation profiles in different cell types.

## Author Summary

Exons that code for protein can also have additional functions, such as regulating gene transcription through enhancer activity. Here, we changed every nucleotide in three different exons that also function as enhancers, and examined their enhancer activity to test whether nucleotide changes in these exons can affect both the protein sequence and enhancer function. We found that mutations with a significant effect on enhancer function can reside both in regions that change the protein sequence (non-synonymous) and regions that do not change it (synonymous). When we conducted a similar analysis in a different cell type, we observed a difference in the nucleotide changes that cause a significant effect on enhancer activity, suggesting that the enhancer functional units can differ between tissues.

## Introduction

Protein coding sequences have been shown to contain additional functional information such as splicing [1], [2], mRNA stability [3], microRNA target sites [4], and transcriptional enhancer activity [5]–[10]. Furthermore, by analyzing various genomic datasets, numerous exons were shown to interact with promoter and enhancer-like regions suggesting that they are involved in alternative splicing, chromatin structure and gene regulation [2]. A study that analyzed enhancer-associated ChIP-seq datasets found that, on average, 7% of peaks overlap coding exons [5], suggesting that numerous eExons are embedded in mammalian genomes. Furthermore, functional characterization of potential eExons in zebrafish [9] and mice [5] found that over half of them are functional developmental enhancers. In addition, it was shown that eExons can regulate the gene they reside in [6]–[10] and also nearby genes [5], suggesting that mutations in these exons could lead to phenotypes that are not due to their protein function.

The functional consequence of non-synonymous mutations is well established [11]. Synonymous mutations have also been shown to have a functional effect, for example causing disease by improper splicing [12]. However, whether mutations in coding exons can lead to phenotypes by impacting enhancer function is not well known. A recent study demonstrated that human chromosomal abnormalities encompassing two eExons, *DYNC1I1* exon 15 and 17, that regulate developmental limb expression of two neighboring genes, *DLX5* and *DLX6*, could cause split hand and foot malformations [5], similar to a *DLX5* coding mutation [13]. Transcription factors involved in gene regulation were suggested to bind to coding protein sequences and are thought to influence codon choice and, consequently, protein evolution [14]. However, the functional consequence of point mutations in eExons has yet to be determined.

Here, we identified several functional liver eExons and used massively parallel reporter assays (MPRA) [15]–[19] to determine the functional consequences of all possible nucleotide substitutions in three of them. We found that synonymous and non-synonymous mutations have a similar effect on enhancer activity and deleterious enhancer mutations tend to overlap liver-associated transcription factor binding sites (TFBS). Using similar MPRA assays in HeLa cells, we show that their mutational profile can vary in different cell types.

## Results

### ChIP-seq analysis of liver eExons

In order to identify functional liver eExons for MPRA, we analyzed liver-associated ChIP-seq datasets for coding exons that have enhancer marks. We analyzed enhancer associated ChIP-seq datasets (H3K4me1, H3K27ac and p300) of human hepatocytes generated in a separate study (Smith RP et al., manuscript in preparation) and in 8 week old mouse liver [20] (see methods). Since these enhancer marks could also identify potential promoters, we excluded the first exons of genes that had enhancer marks from our subsequent analyses, as these exons could potentially be promoters and not necessarily enhancers (Table S1). For the H3K4me1 and H3K27ac ChIP-seq datasets, we found that 18–20% and 9–11% of all peaks overlap coding exons in human hepatocytes and adult mouse liver, respectively. Since the average peak size of these two enhancer marks is rather long (∼2 kb), we also analyzed p300 ChIP-seq datasets, which have a shorter average peak size (∼400 bp). We found that 7% and 6% of all peaks overlap coding exons in human hepatocytes and mouse liver, respectively, (Table S1).

We next wanted to increase our chances of obtaining a functional eExon for our enhancer assays and subsequent MPRA. We thus chose the human hepatocyte p300 ChIP-seq dataset for all subsequent analysis (Figure S1). This was done due to its aforementioned shorter peak size, thus increasing the chances that a substantial exon-peak overlap will be indicative of function, since 80% of all human exons are <200 bp in length [21]. We selected only coding exons where at least 25% of the human hepatocyte p300 ChIP-seq peak overlapped the actual exon. Using transcription factor (HNF4A, FOXA1, FOXA2) ChIP-seq datasets from HepG2 cells [22], our eExon candidates were further filtered for peaks that were bound by at least two of these transcription factors. From the remaining 48 eExon candidates, we selected 15 for individual enhancer assays (Table S2).

### Functional characterization of liver eExons

**Figure 1:**
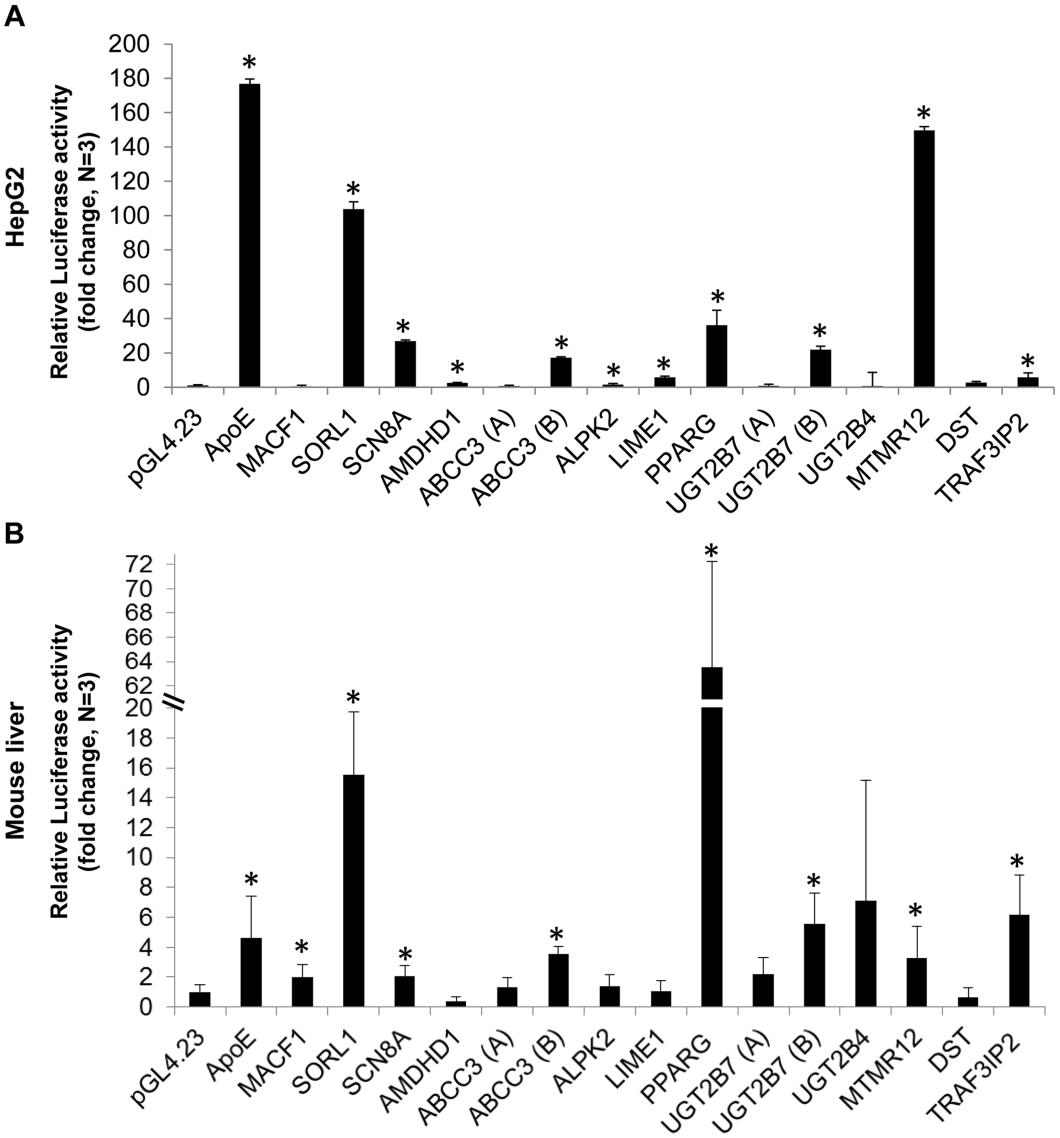
Functional enhancer assays. Bar charts representing the luciferase activity of 15 selected eExon candidates relative to the empty vector (*pGL4.23*) and the apolipoprotein E (ApoE) liver enhancer [50], used as a positive control. Ten exons showed significant luciferase activity in HepG2 cells (A) and eight exons in mouse liver (B), when compared to the empty vector. The results represent the means ± standard deviation of 3–5 independent experiments (*p<0.05; t-test).

To determine whether these exons function as liver enhancers, we tested the fifteen selected candidates for enhancer activity in human hepatocellular carcinoma (HepG2) cells and mouse liver. The selected ChIP-seq exonic peaks were amplified from human genomic DNA and cloned into the *pGL4.23* vector (Promega) that contains a minimal promoter followed by the luciferase reporter gene. The vectors were transfected into HepG2 cells and luciferase activity was measured after 24 hours. Ten out of the fifteen tested exons had significant luciferase activity versus the empty vector (p<0.05; t-test), suggesting that they function as enhancers (Figure 1A). We next tested these sequences in mice using the hydrodynamic tail vein injection [23], [24] and found that eight out of the fifteen exons showed significant liver enhancer activity (Figure 1B; p<0.05; t-test). Combined, seven exons functioned as enhancers in both HepG2 and mouse liver.

### Mutation analysis of three liver eExons using massively parallel reporter assays

Since eExon mutations could alter both protein coding function and enhancer activity, we were interested to assess the effect of single nucleotide variants (SNVs) on enhancer function at single base pair resolution. We selected three eExons with the highest enhancer activity from the mouse enhancer assay for MPRA: 1) exon 17 of sortilin-related receptor, L (DLR class) A repeats containing (*SORL1*) gene, which encodes the YWTD domain of this receptor (also named LR11/SorLA). SORL1 is thought to play a role in endocytosis and sorting and is expressed in the human central nervous system and liver [25], but absent in the mouse liver [26], [27]; 2) exon 2 of TRAF3 interacting protein 2 (*TRAF3IP2*; also known as ACT1) which encodes the Helix-loop-helix (HLH) domain of this protein. TRAF3IP2 is a signaling adaptor protein involved in the regulation of adaptive immunity through the IL-17 pathway [28]–[30] and is expressed in B-cells and liver [31], [32]. 3) exon 6 of the peroxisome proliferator-activated receptor gamma (*PPARG*) gene, which encodes the ligand binding domain of this protein. PPARG is a nuclear receptor that regulates fatty acid storage and glucose metabolism and is expressed in the liver [33].

**Figure 2:**
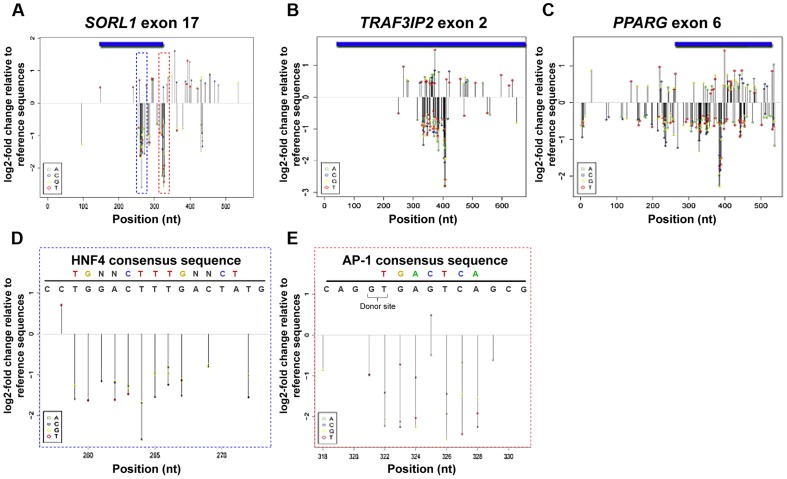
MPRA regulatory profiles of three liver eExons. (A–C) Estimated effect size of all possible nucleotide substitutions based on coefficients from a trivariate (A:green, C:blue, G:yellow, T:red) model are shown for all three liver eExons: (A) *SORL1* exon 17, (B) *TRAF3IP2* exon 2 and (C) *PPARG* exon 6. Effect sizes are shown only for positions where model coefficients had associated *P*-values≤0.01. (D, E) Mutation effect sizes in regions overlapping predicted TFBS in *SORL1* exon 17. A predicted HNF4A site at positions 259–272 (D; blue dashed box) and predicted AP-1 site at positions 322–327 (E; red dashed box) are shown along with the effect size for each possible substitution. The consensus TFBS (top colored motif) and the tested eExon sequence (black) are shown above. In E, SNV 325 G>C that increases the similarity to an AP-1 consensus sequence is shown in bold and the location of the donor splice site is also indicated.

Using MPRA, we systematically dissected the functional consequences of all possible SNVs for these three eExons in the mouse liver (Figure 2). By oligonucleotide synthesis and polymerase cycling assembly, we generated for each eExon a low complexity library (≥10,000) of enhancer mutant haplotypes that diverged from the wild type sequence by ∼2–3% (Table S3). These mutant enhancers, along with 20-bp degenerate tags, were cloned into *pGL4.23* (see experimental procedures). We performed subassembly on each library to determine the full sequence of each enhancer haplotype and its represented tag [34]. Each library was then assayed in mice using the hydrodynamic tail vein injection, livers were harvested after 24 hours and the relative activities of individual haplotypes were measured by sequencing the transcribed tags. Using linear regression analysis, the effect-size of every possible single-nucleotide change on enhancer activity was estimated (see experimental procedures). We found that the functional consequence of most mutations was modest, with ∼18% affecting activity by ≥1.2-fold and ∼4% by ≥2-fold. The majority of SNVs with ≥2 fold change reduced enhancer activity (92%) and only a few SNVs increased activity (8%) (Table S4). To validate our results, we individually tested selected SNVs using the hydrodynamic tail vein assay and found a high correlation with their MPRA profiles (R = 0.94; Figure S2).

We next examined the functional profile of each eExon. In all three eExons, we observed dispersed clusters that had high effect sizes (Figure 2A–C). For example, the first ∼250 bases and the last 120 bases of *SORL1* exon 17 did not have a strong effect on enhancer activity, while nucleotide substitutions at positions 259–328, which are mostly coding sequences, had a significant effect on enhancer activity (Figure 2A). For *TRAF3IP* exon 2, the first ∼330 bases and the last ∼240 bases did not have a significant effect on enhancer activity, while almost all nucleotide substitutions at protein coding positions 333–409 had a significant impact (Figure 2B). *PPARG* exon 6 had clusters of nucleotide substitutions along the entire tested sequence with a significant impact on enhancer activity (Figure 2C). These results point to the existence of discrete regions in these eExons that modulate enhancer activity and also code for important protein domains.

### Enhancer function is integrated with protein function

We next investigated whether the functional regions of the enhancer are subjective to the protein code or impartial to it by analyzing the effect-size of synonymous versus non-synonymous SNVs in these three eExons. We divided the functional SNVs (Fold change (FC) ≥1.2) to synonymous and non-synonymous and compared the effect size of synonymous versus non-synonymous SNVs on enhancer function. We found no significant differences between synonymous and non-synonymous SNVs and their effect-sizes (p-value = 0.67; Fisher test). A similar analysis for just the high functional SNVs (FC≥2) also showed no significant differences (p-value = 0.36; Fisher test). Thus, our results suggest that the essential functional enhancer sequences are intertwined with the protein coding sequences.

### Functional clusters co-localize with transcription factor binding sites

We analyzed the profiles of each eExon to determine whether SNVs that significantly affect enhancer activity overlap with liver-associated TFBS. We found several positions separated by less than ∼6 nucleotides that had significantly correlated effect sizes (P≤0.01) (Figure S3 A–C), suggesting that these clusters might represent TFBS. Indeed, when we analyzed these eExons for liver-associated TFBS, we observed a striking overlap between predicted liver-associated TFBS and these clusters (Table S5; Figure 2D,E). Across the three tested eExons, AP-1 and HNF4A were the most prevalent TFBS overlapping these clusters. For example, a predicted HNF4A binding site at position 259–272 (Figure 2D) and a predicted AP-1 binding site at position 322–327 (Figure 2E) in *SORL1* exon 17, overlap a cluster of SNVs that significantly affect enhancer activity. All the mutations overlapping the AP-1 binding site had a negative effect on enhancer activity, with the notable exception of a variant with a positive effect (325 G>C) that likely increased the AP-1 binding affinity (Figure 2E). In addition, we also observed mutations that generated novel TFBS that increased enhancer activity. For example, in *TRAF3IP2* exon 2, 373G>T, and two neighboring nuclear factor 1(NF-1) binding sites, 393A>T, 399T>A, in *SORL1* exon 17 were created by nucleotide substitutions and subsequently increased enhancer activity (Figure S3D–E).

**Figure 3:**
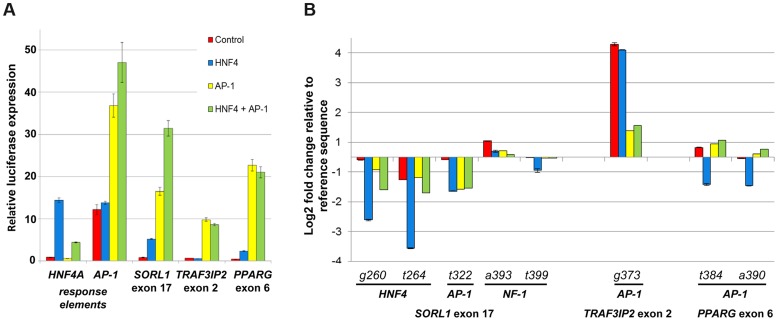
HNF4A and AP-1 alter eExon enhancer activity. (A) All three eExons show enhancer activity in HEK293T cells only when co-transfected with HNF4A and/or AP-1, a heterodimeric protein composed of c-Fos and c-Jun. Co-transfection of both HNF-4 and AP-1 shows an additive effect in *SORL1* exon 17 but not in *TRAF3IP2* exon 2 and *PPARG* exon 6. Both HNF4A and AP-1 response elements show significant luciferase activity when transfected with HNF4A or AP-1 respectively. (B) eExon SNVs that alter AP-1 and HNF4A sites affect enhancer activity in HEK293T cells. For *SORL1* exon 17, SNVs overlapping predicted HNF4A (260G>T, 264G>A) and AP-1 (322T>G) sites had lower luciferase activity in HNF4A and/or AP-1 transfected cells, while SNVs (393A>T, 399T>A) coinciding with a predicted NF-1 TFBS had similar luciferase activity as the reference sequence. For *TRAF3IP2* exon 2, a SNV (373G>T) generating a predicted AP-1 site had higher luciferase activity in control as well as HNF4A and/or AP-1 transfected cells. SNVs 384T>A and 390T>A in *PPARG* exon 6 alter a predicted AP-1 site that did not reduce enhancer activity in AP-1 transfected cells. The luciferase activity results are relative to the Renilla activity and represent the means ± standard deviation of three independent experiments.

To assess whether the binding of AP-1 and HNF4A is affected by these SNVs, we carried out co-transfection assays in HEK293T, a human embryonic kidney cell line where these three eExons are inactive (Figure 3A). Co-transfection of AP-1 and/or HNF4A (see online methods) along with the three enhancer vectors showed significant enhancer activity for all three eExons, suggesting that AP-1 and HNF4A regulate their activity (Figure 3A). We then evaluated whether SNVs that alter AP-1 and HNF4A binding sites could change their enhancer activity. For *SORL1* exon 17, deleterious mutations in predicted HNF4A (260G>T, 264G>A) and AP-1 (322T>G) TFBS reduced enhancer activity when the applicable TF was transfected, while SNVs (393A>T, 399T>A) coinciding with NF-1 had similar enhancer activity as the reference sequence (Figure 3B). A SNV in *TRAF3IP2* exon 2 (373G>T) that generated a predicted AP-1 binding site led to significant enhancer activity both with and without the transfected TFs (Figure 3B). Not all SNVs that altered predicted TFBSs led to the anticipated enhancer activity changes in our assay. For example, SNVs 384T>A and 390T>A in *PPARG* exon 6, that alter an AP-1 TFBS, did not reduce enhancer activity in AP-1 transfected cells (Figure 3B). Combined, our results demonstrate that liver expressed TFs, such as AP-1 and HNF4A, modulate the enhancer activity of these eExons and mutations in their predicted TFBSs could partially explain the effect of SNVs on enhancer activity.

### Tissue-specific regulatory profiles of eExons

To determine whether the distinctive TF repertoire of different cells could modulate enhancer activity, we set out to carry a similar MPRA analysis for all three eExons in a different cell type and compare the results to the regulatory profile from mouse liver. Using available TF ChIP-seq datasets [22], we observed that these three eExons overlap several ChIP-seq peaks in various human cell lines (Figure 2C–E). Of note, *PPARG* exon 6 and *TRAF3IP2* exon 2 specifically overlap AP-1 ChIP-seq peaks in human cervical cancer cells (HeLa) cells. Subsequent luciferase reporter assays showed that all three eExons are active enhancers in HeLa cells (Figure S4A). We thus set out to do a similar MPRA experiment in HeLa cells for all three eExons.

**Figure 4:**
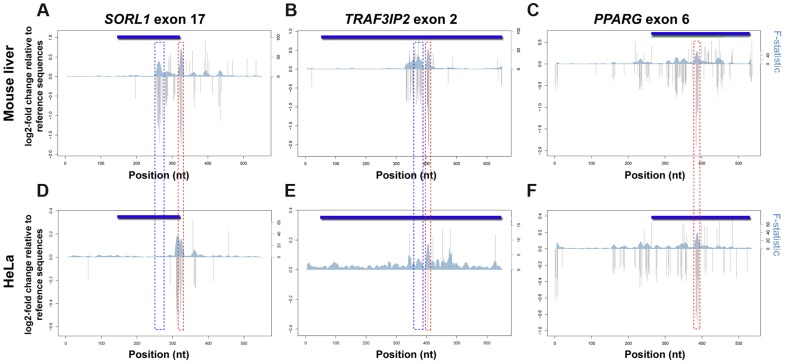
Regulatory profiles of eExons in mouse liver and HeLa cells. Estimated effect sizes of all possible nucleotide substitutions based on coefficients from position variant (gray bars) models for each eExon. Multiple linear regressions with sets of ten adjacent positions as predictors were used to analyze the F-statistic of these models that represent the extent to which the model is predictive of the outcome (blue shadow). Both log2 of fold change relative to reference sequence (left; y-axes) and F-statistic (right; y-axes) are plotted for each eExon in mouse liver (A–C) and HeLa cells (D–F): *SORL1* exon 17 (A,D), *TRAF3IP2* exon 2 (B,E) and *PPARG* exon 6 (C,F). Significant effect mutation clusters with a differential profile between mouse liver and HeLa cells that overlap predicted HNF4A binding sites are illustrated by blue dotted rectangles, while mutation clusters that overlap predicted AP-1 binding sites and remained unchanged in both experiments are marked by red dotted rectangles.

Using MPRA, we dissected the mutational profile of these three eExons and found that the functional consequence of most mutations in HeLa cells was low to modest, with ∼12% affecting activity by ≥1.2-fold and ∼1% by ≥2-fold. Comparison to mouse liver mutation profiles showed that the overall effect size of SNVs (with ≥1.2 fold change) was lower in HeLa cells. The regulatory profiles of *PPARG* exon 6 showed high similarity between mouse liver and HeLa cells, while discrete differences were observed for the other two exons (Figure 4). In *SORL1* exon 17 for example, a deleterious mutation cluster in mouse liver that overlaps an HNF4A TFBS at positions 259–272 (Figure 2D) had no effect in HeLa cells (Figure 4A,D), while another mutation cluster at positions 310–317 had a significant effect on enhancer activity in HeLa but not in mouse liver (Figure S4B). Some functional clusters remained unchanged in both experiments (Figure S4B–D). For example, SNVs at positions 322–327 that overlap an AP-1 binding site (Figure 2E) had a similar profile in both cells with a lower effect size in HeLa. To validate our results, individual SNVs overlapping predicted TFBSs were tested in HeLa and were compared to our previous mouse liver results (Figure S2). Overall, we observed luciferase fold-changes that correlated with their corresponding MPRA results (R = 0.77; see methods). Combined, our results demonstrate that the regulatory profile of an enhancer can change between different cell types.

## Discussion

In this study, we analyzed enhancer-associated ChIP-seq datasets (H3K4me1, H3K27ac, p300) from human hepatocytes and mouse liver and observed that on average ∼6% of peaks overlap coding exons after excluding the first exon. Enhancer assays for 15 selected exons that overlap these peaks showed that 8 and 10 of them are functional enhancers in adult mice and human HepG2 cells, respectively. Our observed ∼50% success rate is in line with previous reports that tested eExons for their developmental enhancer activity [5], [9]. However, it is worth noting that in this study we further selected for potentially positive enhancers by looking at ≥25% exon-peak overlap and additional transcription factor ChIP-seq datasets which probably increased our functional enhancer success rate. Also of note, while human *SORL1* is expressed in the liver [25], mouse *Sorl1* is not expressed in this tissue [26], [27], and the human sequence we used showed mouse liver enhancer activity. Previous work has shown that when human sequences are tested for enhancer activity, even in zebrafish, they can portray regulatory activity even if they do not have homologous sequences in that organism [35], [36].

Using MPRAs, we measured the distribution of effect sizes of all possible SNVs in three liver eExons *in vivo*. The three eExon MPRA displayed comparable functional profiles to previously reported liver enhancer MPRA [17], and similarly show that the majority of SNVs have modest effects on transcriptional activity. Since we tested the ChIP-seq peak which includes not only the exonic sequence but also the adjacent intronic sequences, the distribution of effect sizes allowed us to determine the location of the enhancer core and whether the protein coding sequence is required for enhancer activity. For two eExons, *SORL1* exon 17 and *TRAF3IP2* exon 6, the distribution of SNVs with a significant effect on enhancer activity were specifically clustered in coding sequences that encode important protein domains. In addition, non-synonymous mutations in all three eExons had deleterious effects on enhancer activity, indicating that the genetic code can contain overlapping functional information. Interestingly, the dual function of eExons is not only restricted to protein coding function, as the cluster with the highest impact on enhancer activity in *SORL1* exon 17 overlaps a donor splice site (Figure 2E). Alternative splicing occurs simultaneously with nascent RNA transcription [2] and these eExons could also be splicing regulators. These results suggest that mutations in eExons could lead to multiple phenotypes, both from disruption of the protein function and transcriptional regulation.

Our study has several caveats. One caveat is that we are not testing the enhancer activity of these sequences in their natural setting. Similar to most standard enhancer assays, our study takes out these sequences from their natural settings and places them in front of a minimal promoter that is different than their target promoter. In addition, the transcriptional status of these exons in concert with their enhancer activity is not being assessed. Future studies that create knockin mice to selected exon coding mutations that have a deleterious effect on enhancer activity, as determined by our MPRA, could potentially assay this. We also do not know the target genes that are regulated by these eExons. The genes these exons reside in are expressed in most of the tissues and cell types that we analyzed (Table S2), but this does not necessarily establish that they are their target genes. Furthermore, analysis of available chromatin interaction Hi-C datasets [37], [38] from different cell types (IMR90, hESC) suggest that these eExons interact with their gene promoter region as well as nearby gene promoters and other intergeneic regions (Table S7).

An interesting and still open question is what sort of evolutionary mechanism permitted coding exons to acquire another function as transcriptional enhancers. Two different forces could have been the drivers for their evolutionary constraint: protein function or transcription regulatory function, such as enhancer activity. Regarding protein constraint, previous studies have shown that 70% of amino acids in a protein can be altered while maintaining its structure and function [39]. This suggests that, in addition to synonymous sites, non-synonymous changes could allow for substantial flexibility, accommodating for enhancer function. Comparison between synonymous and non-synonymous SNVs in our study did not find any significant differences in their effect on enhancer activity. Supporting this is a recent study that used genomic deoxyribonuclease I footprinting analysis within synonymous and non-synonymous sequences and found that transcription factor binding could possibly impose a functional constraint of both the regulatory code and amino acid choice [14]. Analysis of synonymous constraint elements (SCE) [40], which are significantly conserved synonymous regions, found that only ∼6% of them overlap exonic human hepatocytes and mouse liver ChIP-seq peaks (Table S6). Four of the 15 tested exons overlap SCE, and *PPARG* exon 6 was the only eExon tested by MPRA to overlap an SCE (Table S2). These results suggest that at least for our selected eExons, a tendency for synonymous constraint was not observed (Table S2). The evolutionary constraint of noncoding enhancers has frequently served as a proxy for functional constraint [41], [42], but studies have also shown that many noncoding enhancers evolved rapidly and that mammalian genomes contain large numbers of evolutionarily young, sometimes species-specific, enhancers [43], [44]. Therefore, TFs that play a role in enhancer activation might not discriminate between coding and noncoding sequences and eExons might not be under additional evolutionary constraint than typical coding sequences.

The activity of enhancers, both coding and noncoding, is regulated by the binding of TFs. The cell-specific TF repertoire regulates enhancer activity levels and depends on motif positioning and larger regulatory context. Techniques such as ChIP-seq can identify the TF binding repertoire of a certain enhancer, but are largely limited in their resolution, technological biases and require a priori knowledge about potential TFs that bind to the enhancer. By testing the regulatory profile of three enhancers in two cell types, we identified specific TFBS overlapping positions that can have a different impact on enhancer activity in another cell type. Moreover, functional positions that do not overlap predicted TFBS could identify novel TFs that control enhancer activity in that cell type. Enhancer sequences can encompass a wide repertoire of TFBS. Our study suggests that a collection of TFs could dictate the activity of enhancers in a specific cell type, while a different collection of TFs could dictate enhancer activity in another cell type.

## Materials and Methods

### Data availability

In addition to raw sequencing reads available in the NCBI Short Read Archive (SRA) under accession SRP044727, a full list of mutations, along with their associated effect sizes and p-values, are provided as Table S5.

### eExon mutation library construction

Enhancer haplotypes were generated from short, doped oligonucleotides using polymerase cycling assembly (PCA) as previously described [17]. Sets of overlapping oligonucleotides for each eExon were designed using DNAWorks [45]. Oligonucleotides were synthesized by Integrated DNA Technologies. All positions corresponding to the enhancer region (except for the flanking primer landing sites on either side of the enhancer) were synthesized using a hand-mix doped at a ratio of 97∶1∶1∶1 (that is, designated base at a frequency of 97%, and every other base at a frequency of 1%). The degenerate tags were first cloned to create a complex library of tagged *pGL4.23* plasmids as described in Patwardhan et al, 2012 [17]. Briefly, the tag oligonucleotide (TAG_OLIGO) was made double-stranded using primer extension in a 50 µl reaction volume with 1× iProof Master Mix, 0.5 µg single-stranded tag oligo, 0.5 µg reverse primer (TAG_EXTEND). The reaction was incubated at 95°C for 3 min, 61°C for 10 min and then 72°C for 5 min. The product was purified using a QIAquick column and eluted in 50 µl EB. It was further subjected to ExoI treatment in 40 µl reaction volume for 1 h at 37°C to degrade any remaining single-stranded DNA, and purified again using QIAquick columns. The resulting double-stranded tag oligo was then cloned into *pGL4.23* at the XbaI site (at 1,799 bp) using standard InFusion (Clontech) protocol. The InFusion reaction was diluted to 100 µl using TE8. 1.5 µl of the diluted cloning reaction was used to transform 50 µl of chemically competent FusionBlue cells (Clontech) using standard protocols. The enhancer haplotypes were then cloned into the tagged *pGL4.23* plasmids using standard InFusion protocol. 2.5 µl of the cloning reaction was used to transform 50 µl of chemically competent cells (Stellar) using standard protocol. For each of the three enhancers, eight transformation reactions were pooled and grown overnight in 50 ml liquid culture at 37°C in a shaking incubator. DNA was extracted using the Invitrogen ChargeSwitch Midi Prep Kit.

### Cell culture and reporter assays

1–2×10^5^ of HepG2, HEK293T and HeLa cells (ATCC) were cultured in 24 well plates overnight using standard protocols and were transfected with 500 ng of the enhancer candidate cloned into *pGL4.23* plasmid, along with 50 ng of the Renilla vector, to correct for transfection efficiency, using X-tremeGENE HP DNA transfection reagent (Roche). After 24 hours, the enhancer activity was measured using the Dual-Luciferase reporter assay (Promega) on a Synergy 2 microplate reader (BioTek Instruments). In HEK293T, the enhancer variant vectors were co-transfected along with 100 ng of HNF4α2 and HNF4α8 expression constructs for HNF4A [46] and c-Fos and c-Jun for AP-1, which is a heterodimer of c-Fos and c-Jun [47]. 500 ng of the luciferase reporter construct pZL.HIV.LTR.AI-4 [48], that contains four tandemerized HNF4 response elements was used as a positive control for HNF4A transfection and 500 ng of *pGL4.13* (Promega) that contains an AP-1 responsive element was used as a positive control for c-Fos and c-Jun transfections [49]. For MPRA libraries, 1–2×10^6^ HeLa cells were cultured on 10 mm culture dishes overnight using standard protocols and transfected with 10 µg of the constructed library. After 24 hours, cells were harvested and total RNA was purified using the RNAeasy maxi prep kit (Qiagen) and subjected to mRNA selection (Oligotex, Qiagen) following the manufacturer's protocols.

### Hydrodynamic tail vein enhancer assay

For the hydrodynamic tail vein assay, each tested sequence or MPRA library, cloned in the *pGL4.23*[*luc2*] vector, were injected (10 µg) alongside 2 µg of *pGL4.74*[*hRluc*/TK] vector to correct for injection efficiency, into five CD1 mice (Charles River Laboratories) using the *Trans*IT EE hydrodynamic gene delivery system (Mirus Bio LLC) according to the manufacturer's protocol. Negative (empty *pGL4.23*[*luc2*]) and positive (*ApoE* liver enhancer [50] controls (*n* = 5) were also injected in parallel at each injection date/experiment. After 24 h, the mice were euthanized, livers harvested and used to make RNA in order to sequence transcribed tags (MPRA libraries) and also to measure luciferase activity (MPRA libraries and individual constructs). Approximately 1gr of mouse liver was used to purify total RNA using the RNAeasy maxi prep and 500 µg were used to select for mRNA. For luciferase measurements, livers were homogenized in passive lysis buffer (Promega), followed by centrifugation at 4°C for 30 min at 14,000 rpm. Firefly and *Renilla* luciferase activity in the supernatant (diluted 1∶20) were measured on a Synergy 2 microplate reader in replicates of six for each liver, using the Dual-Luciferase reporter assay system. The ratios for firefly luciferase:Renilla luciferase were determined and expressed as relative luciferase activity. All mouse work was approved by the UCSF Institutional Animal Care and Use Committee protocol number AN100160.

### Sequencing of RNA-derived tags of eExon haplotype libraries

20-bp tags were identified in liver/HeLa mRNA using previously described methods [17]. Sixteen RT-PCR reactions were performed for each of the biological duplicates, which were then multiplexed and sequenced on a GAIIx (Illumina) using a custom sequencing primer (TAG_SEQ_F). Each run was either 20 or 36 cycles with an additional 6 cycles to read the indexing tag using the index sequencing primer (TAG_SEQ_INDEX). For each aliquot, reads were filtered based on the quality scores for the first 20 bases, which correspond to the degenerate tag. The corresponding number of reads per each tag was counted and only tags that were supported by at least ten reads were used for further analysis.

### Associating eExon haplotypes with tags

eExon haplotypes were associated with tags as previously described [17]. Briefly, 1,000 bp segments separating eExon haplotypes and tags on the *pGL4.23* plasmid were excised by digesting with HindIII, which cuts both the 3′ of the eExon haplotype, and 5′ of the tag. The digested plasmids were purified and recircularized using intramolecular ligation (T4 DNA Ligase from NEB), resulting in the tag being adjacent to the 3′ end of the eExon haplotype. The region spanning the eExon haplotype and tags was amplified from recircularized plasmids by PCR with the forward primer targeting the region immediately 5′ of the eExon haplotype (eEXON_COMMON_F) and the reverse primer targeting the region immediately 3′ of the tag (TAG_PE_R). The amplicons were then subjected to the subassembly protocol as conceptually described in Hiatt et al. [34]. The random fragmentation step was carried out using the Nextera Tn5 transposase (Illumina) instead of mechanical shearing. The Nextera reaction was purified using MinElute column (Qiagen) and size-selected by PAGE. The size selected fragments were subjected to PCR in 25 ul reaction volume with 1× KapaHiFi Hot Start Ready Mix (Kapa BioSystems), 0.5× SYBR Green I, 20% of the size-selected product and each of the primers, Nextera Adapter 1 and TAG_PE_R at 0.3 uM final concentration. Thermal cycling was carried out using BioRad Mini Opticon System using the following program: 95°C for 3 mins; and then 30 cycles of 98°C for 20 sec, 65°C for 15 sec, 72°C for 15 sec. The PCR products were purified using QiaQuick column and then sequenced on a Hi-Seq 2000. Read1 collected 101 bp of the enhancer sequence staring at random breakpoints along the enhancer. Index read collected the 20 bp tag sequence. The reads were then grouped by tag. Reads belonging to each group were then aligned to the wild type enhancer sequence to identify the mutations on the haplotype associated with that tag using custom scripts.

### eExon haplotype effect size analysis

All linear regression analyses were done using the lm() or lsfit() functions available in the R Statistical Package as previously described [17]. To quantity the effect of mutation at any given position on the number of aliquots in which an enhancer haplotype was observed, a separate linear regression model was used for every position along the enhancer, with a single predictor representing whether the given position was wild-type or mutant (univariate model). Similarly, a modified model was used to include the three possible nucleotide substitutions at any position that estimates effect sizes for each position being driven by specific nucleotide substitutions (trivariate model).

For each position in each enhancer, we constructed a linear model to assess the extent to which the presence of a mutation at that position is predictive of a change in the number of RNA aliquots in which the tag associated with an enhancer haplotype was observed. This is effectively a proxy for its impact on transcriptional activation, *i.e.* “effect size”. Specifically, we use the term “effect size” to describe the log2 fold change in the predicted transcriptional activity, as measured by the number of RNA aliquots in which a specific haplotype appeared, relative to the wild-type. For each of the three enhancers, we first calculated effect sizes separately on the data from each mouse (16 RNA aliquots per mouse). The effect sizes for these biological replicates were highly correlated (Tail-vein: *SORL1* exon 17: r = 0.88, *PPARG* exon 6: r = 0.91, *TRAF3IP2* exon 2: r = 0.92; HeLa: *SORL1* exon 17: r = 0.85, *PPARG* exon 6: r = 0.85, *TRAF3IP2* exon 2: r = 0.8). Based on this high reproducibility and to increase resolving power, we performed all subsequent analyses after combining data across both replicates for each enhancer.

### Estimation of effect size of mutation at each position along the enhancer (univariate model)

To quantity the effect of mutation at any given position on the number of aliquots in which an enhancer haplotype was observed, we built a separate linear regression model at every position along the enhancer, with a single predictor representing whether the given position was wild-type or mutant (Table S3). The predictor was thus a binary variable representing presence (1) or absence (0) of a mutation at that position.
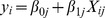
where,

*y_i_* = number of aliquots in which the *i*th haplotype was observed (referred to as aliquot counts),

*X_ij_* = 1 if position *j* was mutant and 0 if position *j* was wild-type in the *i*th haplotype.

To facilitate comparison between positions and between enhancers, we calculated the “effect size” of mutation at a position *j* as:
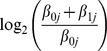
All linear regression analyses were performed using the lm() or lsfit() functions available in the R Statistical Package. The p-value reported by the model for 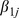 was used to judge whether the effect size was significant.

### Estimation of effect size of each specific nucleotide change at each position along the enhancer (trivariate model)

To explore whether the estimated effect sizes for each position were being driven by specific nucleotide substitutions, we modified the model just described to include three predictors, each representing one of the three possible nucleotide substitutions at that position. The factors were set up as binary variables representing the presence (1) or absence (0) of the particular change at that position.
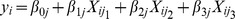
Effect sizes were then calculated from the coefficients produced by the models as follows (for *k* = 1,2,3):
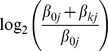
The p-value reported by the model for 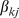 was used to judge whether the effect of a given nucleotide substitution at a given position was significant.

### TFBS identification

Putative TFBSs were identified using MATCH [51] to search for motifs in TRANSFAC Release 2010.3 [52]. MATCH was run independently on each individual sequence with default parameters.

### Computing TF mark scores

For each position with an observed nucleotide substitution, a 51 bp segment of DNA centered at the position was used for a Position Specific Scoring Matrix (PSSM) scan. PSSMs for a subset of liver expressed TFs (Table S5) were obtained from the publicly available JASPAR and TRANSFAC databases. For each PSSM of length L, a ‘mark score’ was calculated for all subsequences of length L within the 51 bp DNA segment that overlapped the central position. A mark score for the reference subsequence (S_r_) and the mutant subsequence (S_m_) were calculated as:
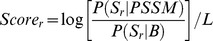


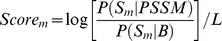
Where B is the background distribution of nucleotides in the genome. TF motifs were only considered if either Score_r_ or Score_m_ was greater than the relative entropy score of the TF. Finally, the TF with the largest absolute score change between Score_r_ and Score_m_ is listed in Table S5. The relative entropy is defined as:
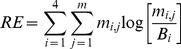
where *m_i,j_* is entry in row I and column j of the PSSM.

## Supporting Information

Figure S1 Computational pipeline for eExon functional assay selection.
(TIF)

Figure S2 Validation of eExons SNVs with strong MPRA effect sizes in mouse liver and HeLa cells. The mutation effect sizes are plotted (log2 fold-change in expression of mutant versus wild-type) for five *SORL1* exon 17 variants, two *TRAF3IP2* exon 2 variants and three *PPARG* exon 6 variants. Each variant was individually injected into five mice or transfected into HeLa cells, and luciferase activity was measured 24 hours post transfection. Effect sizes were compared to those from the mouse liver and HeLa MPRAs and correlated with luciferase activity in the hydrodynamic tail vein (R = 0.942) and HeLa transfection (R = 0.77) assays. The lines above the bars represent the 95% confidence interval. It is worth noting that the MPRA effect sizes of *SORL1* exon 17 (except t322) were not significant and were excluded from the correlation analysis.
(TIF)

Figure S3 Mutational effect analysis of nearby SNV positions and novel TFBS created by these mutations. (A–C) To assess the effect size similarity of mutations at nearby positions in each enhancer, we summed the absolute difference between effect sizes at all positions separated by a fixed “lag” distance. We then recalculated this quantity 1000 times after randomly permuting the effect sizes. We obtained a p-value by calculating the fraction of times that the quantity computed on the permuted effect sizes was at least as small as the quantity computed on the real data. This was repeated for a range of values with varying lag distance. The p-value is plotted here as a function of the lag distance. Positions separated by ∼5 nucleotides or fewer show substantially similar effect sizes (p<0.01) across all three enhancers assayed: *SORL1* exon 17(A), *TRAF3IP2* exon 2 (B) and *PPARG* exon 6 (C). (D) A SNV 373G>T (red rectangle) in *TRAF3IP2* exon 2 that created a predicted AP-1 site is plotted along the effect sizes and AP-1 consensus sequence. (E) Two SNVs, 393A>T, 399T>A, in *SORL1* exon 17 (black rectangles) that create two predicted neighboring nuclear factor 1(NF-1) sites are plotted along the effect sizes and NF-1 consensus sequence.
(TIF)

Figure S4 Enhancer activity of eExons in HeLa cells and their mutation correlation analysis compared to mouse liver. (A)The luciferase activity of each eExon in HeLa cells is compared to the negative control (*pGL4.23*). All three eExons showed significant enhancer activity (p-value≤0.01; t-test). Luciferase levels are relative to Renilla activity and lines represent the means ± standard deviation of three independent experiments. (B–D) MPRA activity profiles for *SORL1* exon 17 (B), *TRAF3IP2* exon 2 (C) and *PPARG* exon 6 (D) were standardized by their mean and standard deviation using the R ‘scale’ function. For each nucleotide, the difference between the HeLa and mouse liver effect size was plotted. The red line represents a smoothing function of the data using Loess regression in R and red peaks represent position clusters with differential effect sizes. Significant effect mutation clusters with a differential profile between liver and HeLa cells that overlap predicted HNF4A (blue dotted rectangles) and AP-1 (red dotted rectangles) sites are also indicated.
(TIF)

Table S1 Analysis of ChIP-seq enhancer-associated datasets for exon overlap.
(PDF)

Table S2 Exons tested for enhancer activity.
(XLSX)

Table S3 eExon library characteristics.
(XLSX)

Table S4 Mutation effect size summary.
(PDF)

Table S5 Summary of all SNVs mutated and analyzed through MPRA.
(XLSX)

Table S6 ChIP-seq peaks overlapping synonymous constraint elements (SCEs).
(PDF)

Table S7 Chromatin interactions for the three eExons as detected by Hi-C in various cell lines.
(XLSX)
