# Supplementary figures and images for: Systematic Dissection of Coding Exons at Single Nucleotide Resolution Supports an Additional Role in Cell-Specific Transcriptional Regulation
Source: PLoS Genet. 2014 Oct 23;10(10):e1004592. doi: 10.1371/journal.pgen.1004592 (PMC4207465; doi:10.1371/journal.pgen.1004592)

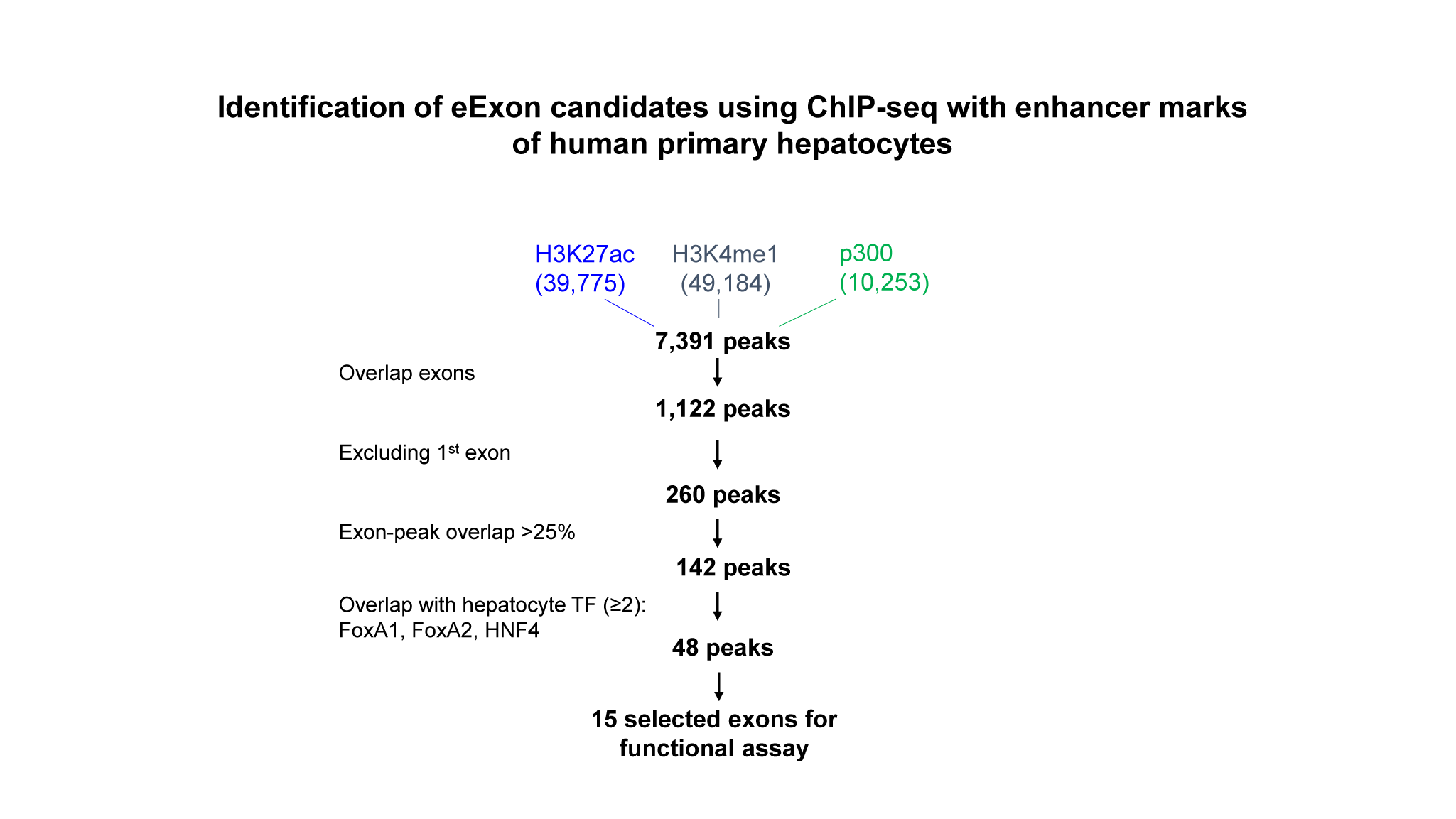

Supplement: Figure S1 — Computational pipeline for eExon functional assay selection. (TIF) [file pgen.1004592.s001.tif]

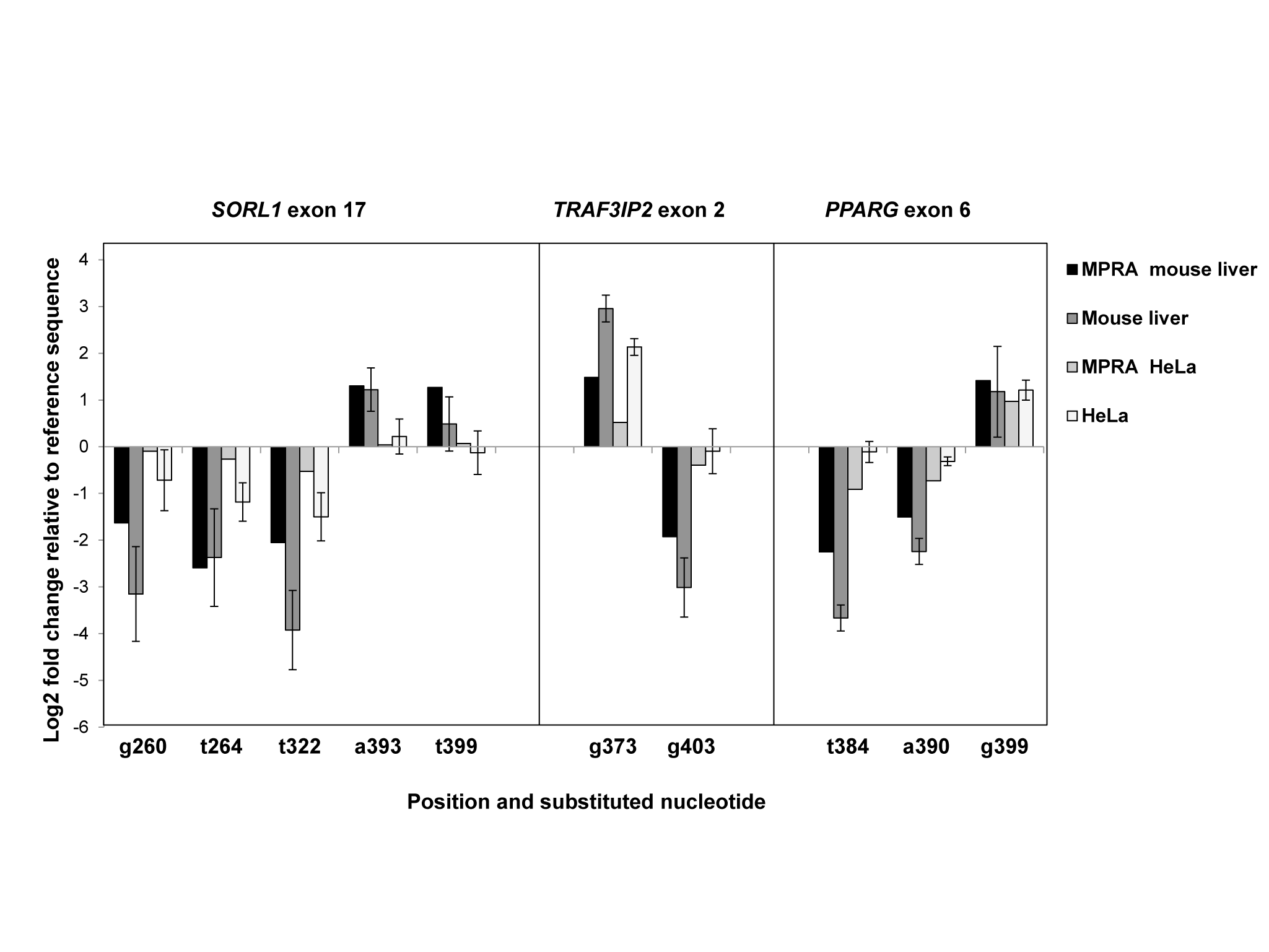

Supplement: Figure S2 — Validation of eExons SNVs with strong MPRA effect sizes in mouse liver and HeLa cells. The mutation effect sizes are plotted (log2 fold-change in expression of mutant versus wild-type) for five SORL1 exon 17 variants, two TRAF3IP2 exon 2 variants and three PPARG exon 6 variants. Each variant was individually injected into five mice or transfected into HeLa cells, and luciferase activity was measured 24 hours post transfection. Effect sizes were compared to those from the mouse liver and HeLa MPRAs and correlated with luciferase activity in the hydrodynamic tail vein (R = 0.942) and HeLa transfection (R = 0.77) assays. The lines above the bars represent the 95% confidence interval. It is worth noting that the MPRA effect sizes of SORL1 exon 17 (except t322) were not significant and were excluded from the correlation analysis. (TIF) [file pgen.1004592.s002.tif]

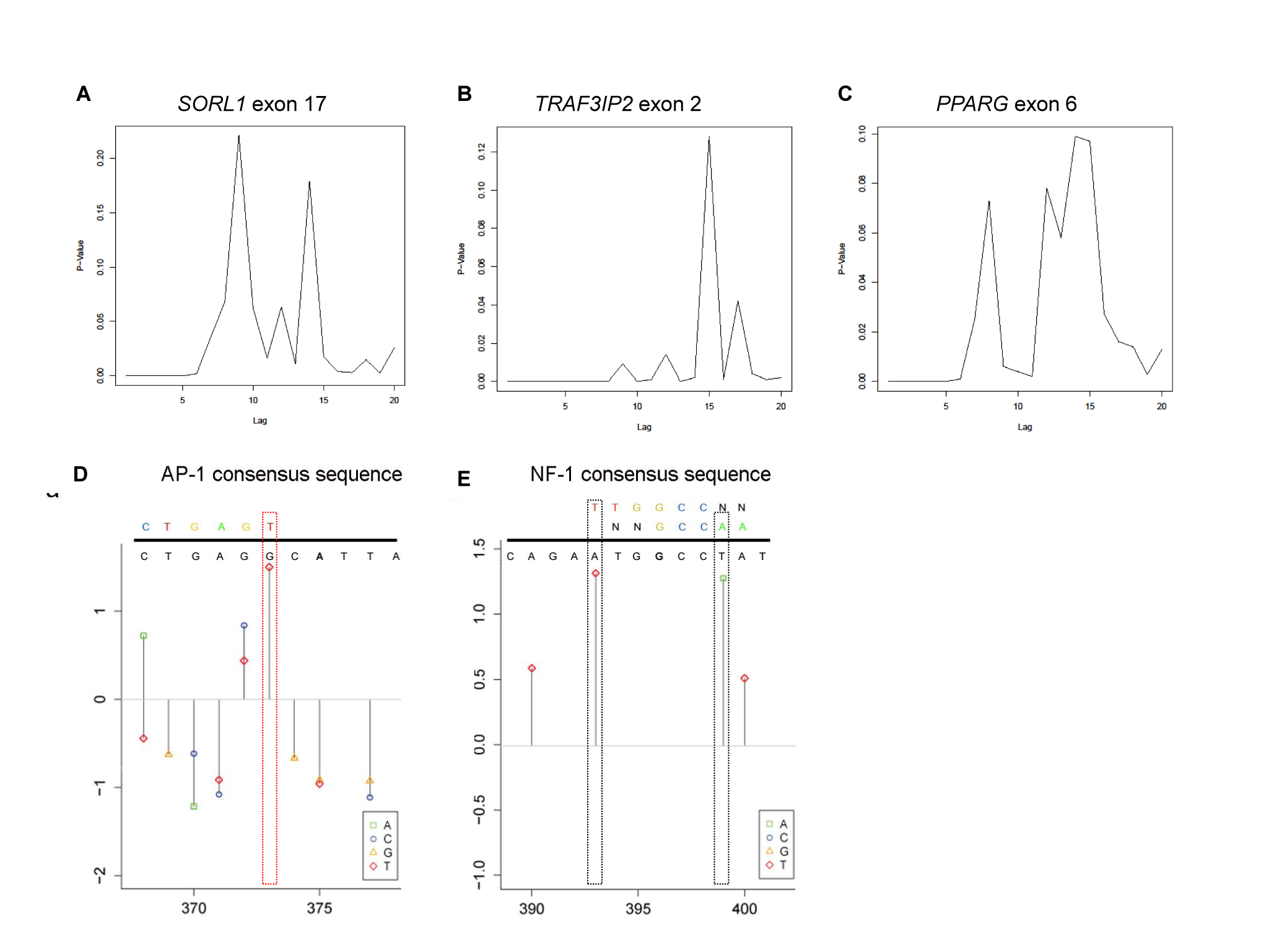

Supplement: Figure S3 — Mutational effect analysis of nearby SNV positions and novel TFBS created by these mutations. (A–C) To assess the effect size similarity of mutations at nearby positions in each enhancer, we summed the absolute difference between effect sizes at all positions separated by a fixed “lag” distance. We then recalculated this quantity 1000 times after randomly permuting the effect sizes. We obtained a p-value by calculating the fraction of times that the quantity computed on the permuted effect sizes was at least as small as the quantity computed on the real data. This was repeated for a range of values with varying lag distance. The p-value is plotted here as a function of the lag distance. Positions separated by ∼5 nucleotides or fewer show substantially similar effect sizes (p<0.01) across all three enhancers assayed: SORL1 exon 17(A), TRAF3IP2 exon 2 (B) and PPARG exon 6 (C). (D) A SNV 373G>T (red rectangle) in TRAF3IP2 exon 2 that created a predicted AP-1 site is plotted along the effect sizes and AP-1 consensus sequence. (E) Two SNVs, 393A>T, 399T>A, in SORL1 exon 17 (black rectangles) that create two predicted neighboring nuclear factor 1(NF-1) sites are plotted along the effect sizes and NF-1 consensus sequence. (TIF) [file pgen.1004592.s003.tif]

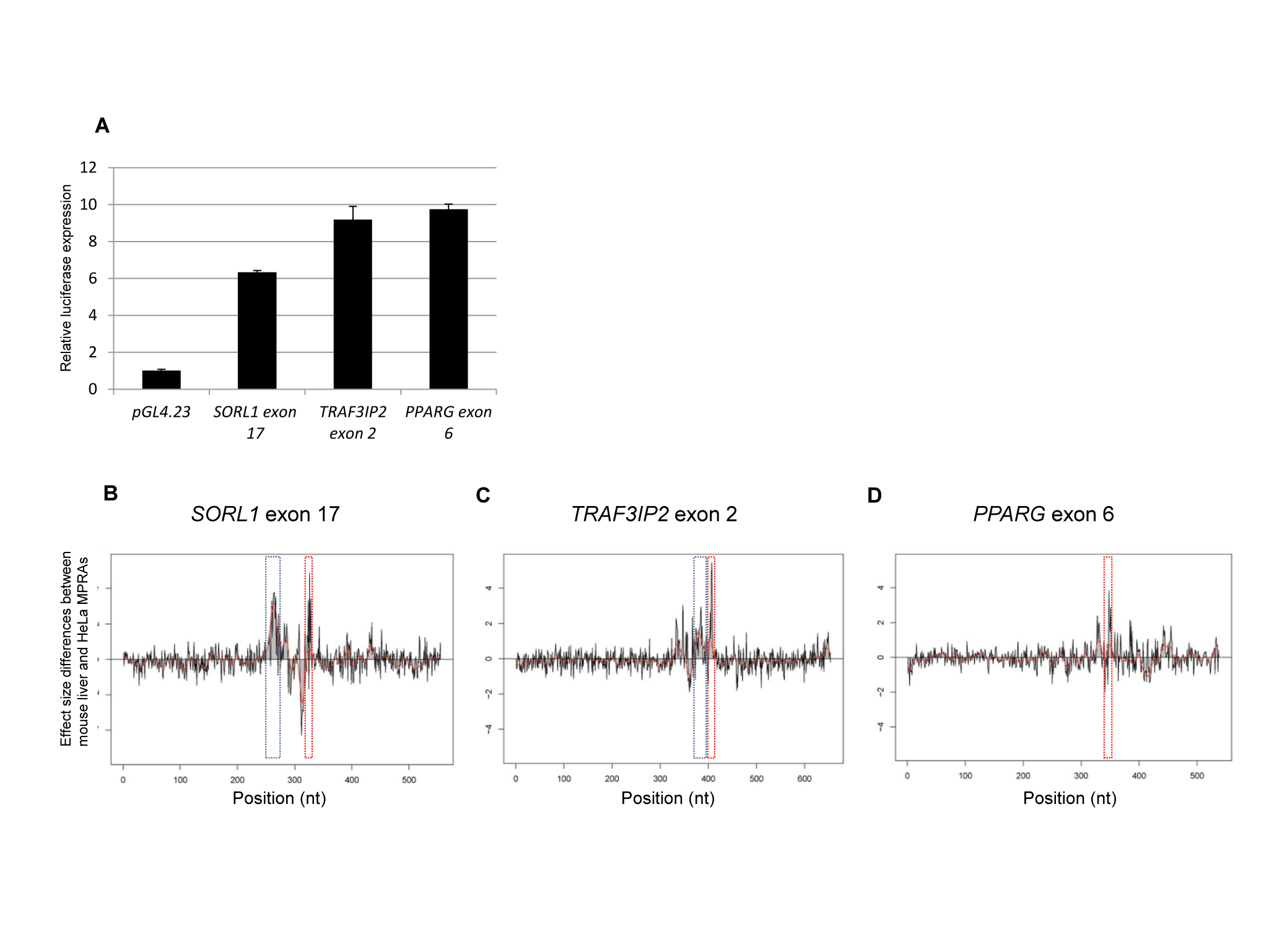

Supplement: Figure S4 — Enhancer activity of eExons in HeLa cells and their mutation correlation analysis compared to mouse liver. (A)The luciferase activity of each eExon in HeLa cells is compared to the negative control (pGL4.23). All three eExons showed significant enhancer activity (p-value≤0.01; t-test). Luciferase levels are relative to Renilla activity and lines represent the means ± standard deviation of three independent experiments. (B–D) MPRA activity profiles for SORL1 exon 17 (B), TRAF3IP2 exon 2 (C) and PPARG exon 6 (D) were standardized by their mean and standard deviation using the R ‘scale’ function. For each nucleotide, the difference between the HeLa and mouse liver effect size was plotted. The red line represents a smoothing function of the data using Loess regression in R and red peaks represent position clusters with differential effect sizes. Significant effect mutation clusters with a differential profile between liver and HeLa cells that overlap predicted HNF4A (blue dotted rectangles) and AP-1 (red dotted rectangles) sites are also indicated. (TIF) [file pgen.1004592.s004.tif]
