# Supplementary material for: Systematic Dissection of Coding Exons at Single Nucleotide Resolution Supports an Additional Role in Cell-Specific Transcriptional Regulation
Source: PLoS Genet. 2014 Oct 23;10(10):e1004592. doi: 10.1371/journal.pgen.1004592 (PMC4207465; doi:10.1371/journal.pgen.1004592)
Supplement: Table S1 — Analysis of ChIP-seq enhancer-associated datasets for exon overlap. (PDF) [file pgen.1004592.s005.pdf]

Table S1. Analysis of ChIP-seq enhancer-associated datasets for exon overlap.

Human hepatocytes

	Total ChIP-seq peaks		ChIP-seq peaks overlapping all exons			ChIP-seq peaks overlapping coding exons			ChIP-seq peaks overlapping coding exons, excluding the 1st exon		
**Antibody**	**Peaks**	**Average peak width**	**eExon peaks**	**% from total**	**Average peak width**	**eExon peaks**	**% from total**	**Average peak width**	**eExon peaks**	**% from total**	**Average peak width**
H3K4me1	49,185	5,001	16,874	34%	6,576	14,738	30%	6,751	8,836	18%	5,991
H3K27ac	39,776	5,503	19,002	48%	6,749	16,240	41%	6,981	8,098	20%	6,073
p300	10,253	377	2,479	24%	389	1,333	13%	404	704	7%	398
Overlapping peaks for all 3 marks	7391	390	1122	14%	406	484	7%	417	260	4%	417

Adult mouse liver 8 weeks

	Total ChIP-seq peaks		ChIP-seq peaks overlapping all exons			ChIP-seq peaks overlapping coding exons			ChIP-seq peaks overlapping coding exons, excluding the 1st exon		
**Antibody**	**Peaks**	**Average peak width**	**eExon peaks**	**% from total**	**Average peak width**	**eExon peaks**	**% from total**	**Average peak width**	**eExon peaks**	**% from total**	**Average peak width**
H3K4me1	37,244	1,518	7,443	20%	1,886	5,946	16%	1,943	4,270	11%	1,908
H3K27ac	28,367	2,183	12,374	44%	2,598	9,241	33%	2,699	2,451	9%	2,635
p300	3,871	480	1,278	33%	587	719	19%	608	225	6%	553
Overlapping peaks for all 3 marks	1,519	529	347	23%	679	218	14%	700	96	6%	603
