# Supplementary material for: Systematic Dissection of Coding Exons at Single Nucleotide Resolution Supports an Additional Role in Cell-Specific Transcriptional Regulation
Source: PLoS Genet. 2014 Oct 23;10(10):e1004592. doi: 10.1371/journal.pgen.1004592 (PMC4207465; doi:10.1371/journal.pgen.1004592)
Supplement: Table S4 — Mutation effect size summary. (PDF) [file pgen.1004592.s008.pdf]

Table S4. Mutation effect size summary.

**Liver (FC≥1.2)**	Tested sequence (bp)	exon (bp)	eExon (exon+intron) positions with FC≥1.2 effect size (P≤0.05)						eExon (exon only) positions with effect size of FC≥1.2 (P≤0.05)									
			# of positions	% from total positions	Decreasing enhancer activity positions		Increasing enahncer activity position		overlap exon		synonymous		non-synonymous		stop codon		intronic	
SORL1	555	174	67	12%	39	7%	28	5%	31	46%	9	29%	21	68%	1	3%	36	54%
PPARG	538	268	163	30%	118	22%	45	8%	91	56%	18	20%	65	71%	8	9%	72	44%
TRAF3IP2	654	636	80	12%	50	8%	30	5%	80	100%	17	21%	59	74%	4	5%	0	0%
Average				18%		12%		6%		67%		23%		71%		6%		

**Liver (FC≥2)**	Tested sequence (bp)	exon (bp)	eExon sequence (exon+intron) positions with effect size of FC≥2 (P≤0.05)						exon sequence positions with effect size of FC≥2 (P≤0.05)									
			# of positions	% from total positions	Decreasing enhancer activity		Increasing enahncer activity position		overlap exon		synonymous		non-synonymous		stop codon		intronic	
SORL1	555	174	27	5%	24	4%	3	1%	15	56%	4	27%	11	73%	0	0%	12	44%
PPARG	538	268	14	3%	13	2%	1	0%	10	71%	2	20%	7	70%	1	10%	4	29%
TRAF3IP2	654	636	24	4%	23	4%	1	0%	24	100%	3	13%	19	79%	2	8%	0	0%
Average				4%		3%		0%										

**HeLa (FC≥1.2)**	Tested sequence (bp)	exon (bp)	eExon sequence (exon+intron) positions with effect size of FC≥1.2 (P≤0.05)						exon sequence positions with effect size of FC≥1.2 (P≤0.05)									
			# of positions	% from total positions	Decreasing enhancer activity positions		Increasing enahncer activity position		overlap exon		synonymous		non-synonymous		stop codon		intronic	
SORL1	555	174	31	6%	21	4%	10	2%	11	35%	3	27%	8	73%	0	0%	20	65%
PPARG	538	268	95	18%	74	14%	21	4%	50	53%	11	22%	39	78%	4	8%	45	47%
TRAF3IP2	654	636	3	0.5%	3	0.5%	0	0%	3	100%	1	33%	2	67%	0	0%	0	0%
Average				8%		6%		2%		63%		28%		72%		3%		

**HeLa (FC≥2)**	Tested sequence (bp)	exon (bp)	eExon sequence (exon+intron) positions with effect size of FC≥2 (P≤0.05)						exon sequence positions with effect size of FC≥2 (P≤0.05)									
			# of positions	% from total positions	Decreasing enhancer activity		Increasing enahncer activity position		overlap exon		synonymous		non-synonymous		stop codon		intronic	
SORL1	555	174	1	0%	0	0%	1	0%	0	0%	0	0%	0	0%	0	0%	0	0%
PPARG	538	268	4	3%	4	1%	0	0%	3	75%	0	0%	3	100%	0	0%	1	25%
TRAF3IP2	654	636	0	0%	0	0%	0	0%	0	0%	0	0%	0	0%	0	0%	0	0%
Average				1%		0%		0%		25%		0%		33%		0%		
