# Supplementary material for: Systematic Dissection of Coding Exons at Single Nucleotide Resolution Supports an Additional Role in Cell-Specific Transcriptional Regulation
Source: PLoS Genet. 2014 Oct 23;10(10):e1004592. doi: 10.1371/journal.pgen.1004592 (PMC4207465; doi:10.1371/journal.pgen.1004592)
Supplement: Table S6 — ChIP-seq peaks overlapping synonymous constraint elements (SCEs). (PDF) [file pgen.1004592.s010.pdf]

Table S6. ChIP-seq peaks overlapping synonymous constraint elements (SCEs).

Human hepatocytes

	Total ChIP-seq peaks that overlap coding exons (hg19)		Total ChIP-seq peaks that overlap coding exons after LiftOver to hg18		Coding exon ChIP-seq peaks overlapping SCE9 windows		Coding exon ChIP-seq peaks overlapping SCE15 windows		Coding exon ChIP-seq peaks overlapping SCE30 windows	
Antibody	Peaks	Average peak width (bp)	Peaks	Average peak width (bp)	Peaks	% from total	Peaks	% from total	Peaks	% from total
H3K4me1	14,738	6,751	14,732	6,752	2,087	14%	2,230	15%	2,320	16%
H3K27ac	16,240	6,981	16,230	6,976	2,409	15%	2,504	15%	2,568	16%
p300	1,333	404	1,331	402	84	6%	108	8%	115	9%
Overlapping peaks for all 3 marks	484	417	469	401	28	6%	37	8%	35	7%

Mouse liver

	Total ChIP-seq peaks that overlap coding exons		Total ChIP-seq peaks that overlap coding exons after LiftOver to hg18		Coding exon ChIP-seq peaks overlapping SCE9 windows		Coding exon ChIP-seq peaks overlapping SCE15 windows		Coding exon ChIP-seq peaks overlapping SCE30 windows	
Antibody	Peaks	Average peak width (bp)	Peaks	Average peak width (bp)	Peaks	% from total	Peaks	% from total	Peaks	% from total
H3K4me1	5,946	1,943	1,099	1,561	232	21%	266	24%	253	23%
H3K27ac	9,241	2,699	1,219	2,164	250	21%	268	22%	275	23%
p300	719	608	310	631	31	10%	29	9%	27	9%
Overlapping peaks for all 3 marks	218	700	81	728	5	6%	5	6%	6	7%
